# Fats and Cancer

**Published:** 1990-03

**Authors:** D. E. Khoo, N. A. Habib

**Affiliations:** Dept. of Surgery, Bristol Royal Infirmary; Dept. of Surgery, Bristol Royal Infirmary


### Fats and Cancer

**D. E. Khoo FRCS, N. A. Habib ChM FRCS**
Dept. of Surgery, Bristol Royal Infirmary

The subject of healthy eating is contentious. if you were to accept all the rumoured findings relating diet to cancer, you could be forgiven for having the impression that all substances taken in the diet were hazardous to health. But in the relationship between dietary fat and cancer, it would appear that a low fat intake is advisable although, as we hope to discuss below, dietary advice cannot be too dogmatic concerning specific fats.

Many dietary factors are implicated in cancer. In the first place, amongst the seemingly harmless substances we consume such as black pepper, mushrooms, celery, parsnips, toast, caffeine and alcohol, to name a few, are substances which may be initiators of carcinogenesis[1]. Calories and fats, on the other hand, appear to be cancer promoters[2]. Epidemiological studies have linked the *total* fat available to mortality and incidence of cancers in selected populations[3,4,5,6]. Increased dietary fat intake was postulated as the cause of the increase in breast cancer incidence in Japanese moving from Japan to America[7]. There are obvious flaws in these studies because individual dietary practices may not actually reflect the total fat available or consumed within a nation. Case control studies in which dietary patterns of patients with colonic and breast cancer were analysed and compared with age and sex match controls showed that dietary fat intake was strongly correlated with total fat intake and saturated fat was the most important single independent variable although monounsaturated oleic acid and polyunsaturated fats were also potent independent factors[8,9]. These studies are perhaps limited by the fact that specific fats are not found in isolation within any particular dietary component.

In contrast to the epidemiological data, however, laboratory studies in animals show quite clearly that polyunsaturated fat in the diet is a much more potent promoter of tumour incidence that saturated fat. Because of the increased calorific value of fat compared with carbohydrate, early investigators examined the effect of calories on tumour promotion finding that calorie restriction has a potent negative effect on tumour promotion[10]. However, it was subsequently shown that dietary fat *per se* had an independent promoting effect[11]. Others have since shown that transplantable[12,13] and carcinogen-induced[14,15] cancers of the breast and carcinogen-induced neoplasms of the colon[16] and preneoplastic lesions of the pancreas[17] may be promoted by a high dietary fat intake. In all cases, for the same overall content of fat in diet, polyunsaturated fats have a greater cancer promoting effect than saturated fats. The discrepancy between this observation and the fact that no specific fat is incriminated alone in epidemiological studies has been explained by Carroll who appeared to show that once the minimum essential fatty acid are supplied in the diet, the overall tumour promotion is the same for saturated and unsaturated fats[18]. Other groups have failed to confirm this finding[19,20] and indeed Carroll's group has not been able to confirm this result in one subsequent investigation[21]. It would appear also, that although dietary polyunsaturated fats promote tumours, this is not a consistent effect of saturated fats whose effect may be the same as a low fat diet.

The mechanism of tumour promotion by fat remains in dispute. For breast cancer a hormonal basis has been suggested[22,23]. Others have suggested that dietary essential fatty acids increase the body stores of dienoic eicosanoids leading to prostaglandin synthesis. A supporting observation is that tumour promotion by dietary polyunsaturated fats can be inhibited by administering indomethacin which inhibits the synthesis of prostaglandins from 20-Carbon fatty acids. It is perhaps more likely that ingested fats are incorporated into cell membranes and thus altering membrane fluidity and the consequent ability of the cell to divide and perform metabolic functions[24]. One recent investigation showed that cell-cell cooperation was inhibited by oleic acid and this may increase the ability of malignant cells to divide[25].

A relatively new area of interest, however, is the investigation of the effects on tumour growth of specific fatty acids rather than lipids. Dietary studies are weakened by the fact that fatty acids are modified during first-pass metabolism, and moreover the fatty acid content of oil-enriched diets is heterogeneous. As discussed above, dietary oils rich in polyunsaturated and monounsaturated fatty acids promote tumour growth. Perhaps more significant is that other fatty acids may inhibit tumour growth. Diets rich in fish oils (n-3 fatty acids) have been found to inhibit tumour growth in primary colonic[26,27], and pancreatic[28] carcinogenesis in rats. Furthermore, it is reported that human breast carcinoma cell lines may also be inhibited in nude mice by a diet high in fish oil[29].

Our work, by contrast, involves the role of one particular fatty acid which seems to inhibit tumour growth. This is stearic acid, a saturated fatty acid which contains 18 carbon atoms. In cell culture, it selectively inhibits rat mammary tumour cells in direct contrast to promotion caused by mono and polyunsaturated fatty acids[30,31]. Using clonogenic assay (as established method of assessing antineoplastic drugs) stearic acid selectivity inhibited a number of tumour cell lines[32]. Recently, in an investigation into the mitogenic activity of faecal diglycerides on human adenoma cell in primary culture, it was found that if a diglyceride contained even a single stearic acid residue mitogenesis was completely inhibited[33]. The yield and incidence of spontaneous mammary carcinomas in mice was inhibited by stearic acid the diet[34], and carcinogen-induced mammary[32] and colonic[35] carcinomas in rats were inhibited by injected stearic acid. Transplantable mammary carcinomas in rats were also inhibited by stearic acid analogues iodostearic and sterculic acid, when given by injection[36], and even a human transitional cell carcinoma line in nude mice was inhibited by sterculic acid[37]. It seems that parenteral or delayed-release administration is necessary for this effect; simple oral administration is ineffective presumably because of the metabolism of fatty acids in the upper gut and liver. In Bristol, the clinical efficacy of iodostearic acid is being investigated in advanced human cancers with some evidence of benefit. The advantage of fatty acids appears to be low toxicity but the need for a formulation which bypasses the upper gut creates practical difficulties.

In conclusion, it appears that fatty acids may have a role to play in cancer therapeutics. As yet dietary manipulation is of no proven benefit in the clinical situation although the evidence points to the possibility that low fat diets may be a form of primary prevention in cancer. We would not, of course, advocate a change in diet to saturated fat as there is clear evidence that saturated fat is implicated in the genesis of atheroma. Some fatty acids such as stearic acid analogues, given parenterally, may have therapeutic value in patients with irresectable cancers.

### REFERENCES

1. AMES, B. N. (1983) Dietary carcinogens and anticarcinogens. Oxygen radicals and degenerative diseases. *Science*, **221**, 1256–1263.
2. KRITCHEVSKY, D., WEBER, M. M., BUCK, C. L. and KLURFIELD, D. M. (1986) Calories, fat and cancer. *Lipids*, **21**, 272–274.
3. SEGI, M., KURIHARA, M. and MATSUYAMA, T. (1969) Cancer mortality for selected sites in 24 countries. No. 5 (1964–1965). Sendai, Japan: Dept of Public Health, Tohoku University School of Medicine.

4. ARMSTRONG, B. and DOLL, R. (1975) Environmental factors and cancer incidence and mortality in different countries, with special reference to dietary practices. *Int. J. Cancer,* **15**, 617–631.
5. WYNDER, E. L. (1969) Identification of women at high risk for breast cancer. *Cancer*, **24**, 1235–1240.
6. REDDY, B. S. (1981) Dietary fat and its relationship to large bowel cancer. *Cancer Res.*, **41**, 3700–3705.
7. WYNDER, E. L., KAJITANI, T., ISHIKAWA, S., DODO, H. and TAKANO, A. (1969) Environmental factors of cancer of the colon and rectum. II Japanese epidemiological data. *Cancer*, **23**, 1210–1220.
8. JAIN, M., COOK, G. M., DAVIS, F. G., GRACE, M. G., HOWE, G. R. and MILLER, A. B. (1980) A case-control study of diet and colo-rectal cancer. *Int. J. Cancer*, **26**, 757–768.
9. MILLER, A. B., KELLY, A., CHOI, N. W. *et al.*, (1978) A study of diet and breast cancer. *Am. J. Epidemiol.*, **107**, 499–509.
10. TANNENBAUM A. (1942) The genesis and growth of tumours. II. Effects of caloric restriction per se. *Cancer Res.*, **2**, 406–407.
11. TANNENBAUM, A. (1942) The genesis and growth of tumors. III. Effects of a high-fat diet. *Cancer Res.,* **2**, 468–475.
12. HILLYARD, L. A. and ABRAHAM, S. (1979). Effect of dietary polyunsaturated fatty acids on growth of mammary adenocarcinomas in mice and rats. *Cancer Res.*, **39**, 4430–4437.
13. RAO, G. A. and ABRAHAM, S. (1976) Enhanced growth rate of transplanted mammary adenocarcinoma induced in C3H mice by dietaryl inoleate. *J. Nat. Cancer Inst.*, **56**, 431–432.
14. HOPKINS, G. J., WEST, C. E. and HARD, G. C. (1976). Effect of dietary fats on the incidence of 7, 12-dimethylbenz(a)-anthracene-induced tumors in rats. *Lipids*, **11**, 328–333.
15. CARROLL, K. K. and KHOR, H. T. (1971) Effects of level and type of dietary fat on incidence of mammary tumors induced in female Sprague-Dawley rats by 7, 12-dimethylbenz(alpha)-anthracene. *Lipids,* **6**, 415–420.
16. REDDY, B. S. and MAEURA, Y. (1984). Tumour promotion by dietary fat in azoxymethane-induced colon carcinogenesis in female F344 rats: influence of amount and source of dietary fat. *J. Nat. Cancer Inst.*, **72**, 745–750.
17. ROEBUCK, B. D. (1986) Effects of high levels of dietary fats on the growth of azaserine-induced foci in the rat pancreas. *Lipids*, **21**, 281–284.
18. CARROLL, K. K. and HOPKINS, G. J. (1979). Dietary polyunsaturated fat versus saturated fat in relation to mammary carcinogenesis. *Lipids,* **14**, 155–158.
19. CHAN, P-C., FERGUSON, K. A. and DAO, T. L. (1983) Effects of different dietary fats on mammary carcinogenesis. *Cancer Res.*, **43**, 1079–1083.
20. KING, M. M., BAILEY, D. M., GIBSON, D. D., PITHA, J. V. and MCCAY, P. B. (1979) Incidence and growth of mammary tumors induced by 7, 12-dimethylbenz[a]anthracene as related to the dietary content of fat and antioxidant. *J. Nat. Cancer Inst.*, **63**, 657–663.
21. BRADEN, L. M., FAULKING, L. J. and CARROLL, K. K. (1987). Effects of polyunsaturated fish and vegetable oils on mammary tumors and hyperplastic lesions in rats, 488–490.
22. CHAN, P. C., HEAD, J. F., COHEN, L. A. and WYNDER, E. L. (1977). Influence of dietary fat on the induction of mammary tumors by N-Nitrosomethylurea: associated hormone changes and differences between Sprague-Dawley and F344 rats. *J. Nat. Cancer Inst.,* **59**, 1279–1283.
23. CHAN, P. C. and COHEN, L. A. (1974) Effect of dietary fat, antiestrogen, and antiprolactin on the development of mammary tumors in rats. *J. Nat. Cancer Inst.*, **52**, 25–30.
24. BOONSTRA, J., NELEMANS, S. A. O., FEIJEN, A. *et al.*, (1982). Effect of fatty acids on plasma membrane lipid dynamics and cation permeability in neuroblastoma cells. *Biochim. et Biophys. Acta,* **692**, 321–329.
25. AYLSWORTH, C.F., WELSCH, C. W., KABARA, J. J. and TROSKO, J. E. (1987) Effects of fatty acids on gap junctional communication: possible role in tumor promotion by dietary fat. *Lipids,* **22**, 445–454.
26. REDDY, B. S. (1987) Dietary fat and colon cancer: effect of fish oil, 233–237.
27. NELSON, R. L. TANURE, J. C., ANDRIANOPOULOS, G., SOUZA, G. and LANDS, W. E. M. (1987) A comparison of dietary fish body oil and corn oil in experimental colorectal carcinogenesis, 518–522.
28. O'CONNOR, T. P., ROEBUCK, B. D. and CAMPBELL, T. C. (1987) Effect of varying dietary omega-3:omega-6 fatty acid ratio on L-azaserine induced preneoplastic development in rat pancreas, 238–240
29. REITZ, R. C., BORGESEN, C. E., PARDINI, L. and PARDINI, R. S. (1987) Effects of dietary fish oil on the growth of a human mammary carcinoma in the athymic nude mouse and on the activity of the carnitine acyltransferase and on the delta-9 acyl Co-A desaturase, 529–533.
30. WICHA, M. S., LIOTTA, L. A. and KIDWELL, W. R. (1979) Effects of free fatty acids on the growth of normal neoplastic rat mammary epithelial cells. *Adv. Cancer Res.,* **39**, 426–435.
31. DOI, O., DOI, F., SCHROEDER, F., ALBERTS, A. W. and VAGELOS, P. R. (1978). Manipulation of fatty acid composition of membrane phospholipid and its effects on cell growth in mouse LM cells. *Biochim. Biophys. Acta,* **509**, 239–250.
32. HABIB, N. A., WOOD, C. B., APOSTOLOV, K. *et al.*, (1987). Stearic acid and carcinogenesis. *Br. J. Cancer*, **56**, 455–458.
33. FRIEDMAN, E., ISAKSSON, P., RAFTER, J., MARIAN, B., WINAWER, S. and NEWMARK, H. (1989) Fecal diglycerides as selective endogenous mitogens for premalignant and malignant human colonic epithelial cells. *Cancer Res.,* **49**, 544–548.
34. BENNETT, A. S. (1984). Effect of dietary stearic acid on the genesis of spontaneous mammary adenocarcinomas in strain A/ST mice. *Int. J. Cancer,* **34**, 529–533.
35. KELLY, S. B., HABIB, N. A., WOOD, C. B. and WILLIAMSON, R. C. N. (1989) Inhibition of colonic carcinogenesis by stearic acid in rats. *Br. J. Surg.*, **76**, 631.
36. KHOO, D. E., KELLY, S. B., HABIB, N. A., ROTHNIE, N. D., WOOD, C. B. and WILLIAMSON, R. C. N. (1989) The effect of fatty acids on colonic carcinogenesis in vivo. *Cancer Det. Prev.,* **14**, 64.
37. FERMOR, B., HABIB, N. A., KELLY, S. B., WOOD, C. B and WILLIAMSON, R. C. N. (1989) Inhibition of RT112 human transitional cell xenografts by stearic and sterculic acids. *Br. J. Surg.*, **76**, 637.

